# Nano-biomimetic carriers are implicated in mechanistic evaluation of intracellular gene delivery

**DOI:** 10.1038/srep41507

**Published:** 2017-01-27

**Authors:** Mohsen Alipour, Saman Hosseinkhani, Reza Sheikhnejad, Roya Cheraghi

**Affiliations:** 1 Department of Nano biotechnology, Faculty of Biological Sciences, Tarbiat Modares University, Tehran, Iran; 2 Department of Biochemistry, Faculty of Biological Sciences, Tarbiat Modares University, Tehran, 14115-175, Iran; 3 Department of Molecular Biology, Tofigh Daru Co, Tehran, Iran

## Abstract

Several tissue specific non-viral carriers have been developed for gene delivery purposes. However, the inability to escape endosomes, undermines the efficacy of these carriers. Researchers inspired by HIV and influenza virus, have randomly used Gp41 and H5WYG fusogenic peptides in several gene delivery systems without any rational preference. Here for the first time, we have genetically engineered two Nano-biomimetic carriers composed of either HWYG (HNH) or Gp41 (GNH) that precisely provide identical conditions for the study and evaluation of these fusogenic peptides. The luciferase assay demonstrated a two-fold higher transfection efficiency of HNH compared to GNH. These nanocarriers also displayed equivalent properties in terms of DNA binding ability and DNA protection against serum nucleases and formed similar nanoparticles in terms of surface charge and size. Interestingly, hemolysis and cellular analysis demonstrated both of nanoparticles internalized into cells in similar rate and escaped from endosome with different efficiency. Furthermore, the structural analysis revealed the mechanisms responsible for the superior endosomal escaping capability of H5WYG. In conclusion, this study describes the rationale for using H5WYG peptide to deliver nucleic acids and suggests that using nano-biomimetic carriers to screen different endosomal release peptides, improves gene delivery significantly.

Gene therapy has captured the attention of scientists around the world to overcome genetic diseases such as cancers, diabetes, multiple sclerosis etc. for many years. However, nucleic acid-based drugs, including plasmids containing transgenes, SiRNAs, antisense oligonucleotides, aptamers and Ribozymes have failed to be as effective as we hoped in clinical trials^1^,^2^,^3^. Indeed, some extracellular and intracellular barriers impede the bioavailability of these drugs at their site of action^4^. Therefore, a delivery system capable of skipping these barriers, is of great interest and in great demand.

In the last few decades, some viral and none-viral carriers have been developed to improve gene delivery methods. Viral delivery, although highly efficient, raises considerable immunological and safety concerns^5^. Therefore, non-viral carriers including polymeric, metal and bio-based materials are being used for specific delivery of DNA-based drugs and other biomolecules to their targets with much less safety concern^6^,^7^,^8^,^9^. In fact, various targeting moieties on these carriers could increase the local concentration and bioavailability of cargo on the cell surface of the target tissue^10^,^11^,^12^,^13^,^14^,^15^,^16^. Nevertheless, intracellular pathway from cell membrane to nucleus, can act as a rate limiting step in the overall gene delivery process; particularly, extreme electrostatic repulsion between nucleic acids and cell membrane, endosome entrapment, the molecularly crowded cytoplasm and highly organized nucleus membrane that severely decrease the efficacy of most synthetic carriers^4^,^17^.

Among these barriers mentioned above, endosome entrapment is the main intracellular obstacle for gene delivery^18^. Endosomal vesicles are produced and gradually matured to a lysosome as a result of endocytosis. The drop in pH and the activation of nucleases that occur during endosome maturation, leads to the degradation of entrapped nucleic acids^19^. Therefore, the release of nucleic acid cargos prior to endosomal maturation is a critical step in gene delivery, otherwise it will be destined to degradation.

Understanding the nature of endosome maturation process and the viral infection mechanism led to the rationale design of fusogenic peptides^20^. These peptides easily translocate across endosome membrane, similar to their viral source, the Influenza virus (e.g GALA, KALA, RALA, E5, K5, H5WYG, JST1, INF7and CADY) and the Human Immunodeficiency Virus (Gp41, TAT, FP23, HGP and PEP1)^21^,^22^,^23^,^24^,^25^,^26^. Among these endosome release peptides, influenza-derived H5WYG (GLFHAIAHFIHGGWHGLIHGWYG) as a pH sensitive and HIV–derived GP41 (GALFLGFLGAAGSTMGA) as a pH insensitive fusogenic peptide have been extensively studied and used to improve the gene delivery efficacy of different polymeric and lipid based carriers^27^,^28^,^29^,^30^,^31^,^32^,^33^,^34^,^35^. However, these carriers have been used under different conditions in terms of cell lines, peptide concentration, stoichiometric of component and the types of cargo^36^. Therefore, it would be hard to compare the endosome release activity of these fusogenic peptides and rationally select one over the other.

Genetic engineering on the other hand, enables researchers to create flexible nanocarriers with desired bio-inspired functional motifs. In fact, biomimetic nanocarriers can be genetically-programmed to execute multiple functions, including, condensing nucleic acid strands into nanoparticle, escaping from endosome and localization in the cell nucleus^37^,^38^,^39^. We have previously used this technology to develop a safe carrier for nucleic acid delivery into mammalian cells^28^. On the other hand, Firefly (beetle) bioluminescence, due to large quantum yield of light production, has illuminated complex intracellular phenomena and have been utilized in numerous biological investigations^40^,^41^. Therefore integration of bioluminescence phenomena and biomimetic nanocarriers, due to their flexible nature, provide valuable tools for pre-evaluation endosome escaping peptides and other functional motifs for intracellular transportation.

In this study, we have engineered and examined, peptide based nano-biomimetic carriers to evaluate their gene delivery capabilities and to elucidate endosomal release mechanism of H5WYG and Gp41 peptides under the same conditions. For this purpose, we assembled two repeats of histone H1 16 mer peptide as a DNA binding motif (H)^42^ and simian virus 40(SV40) NLS as a nuclear delivery motif (N) that were flanked with either H5WYG (H) or Gp41(G) using one-step genetic engineering technology. These genetically assembled carriers are abbreviated as HNH and GNH (Fig. 1a). We have used the luciferase assay to analyze the activity of endosome release motifs of HNH and GNH carriers. The results were validated by a series of *in vitro* analysis of HNH and GNH, including, electrophoresis mobility, nuclease protection, particle size and charge analysis, uptake rate, and hemolysis. Finally, the structural analysis was performed using fluorescence spectroscopy and Circular dichroism to determine the endosomal release mechanism of these carriers.

## Results

### Design of all-in-one nanocarriers

**Figure 1:**
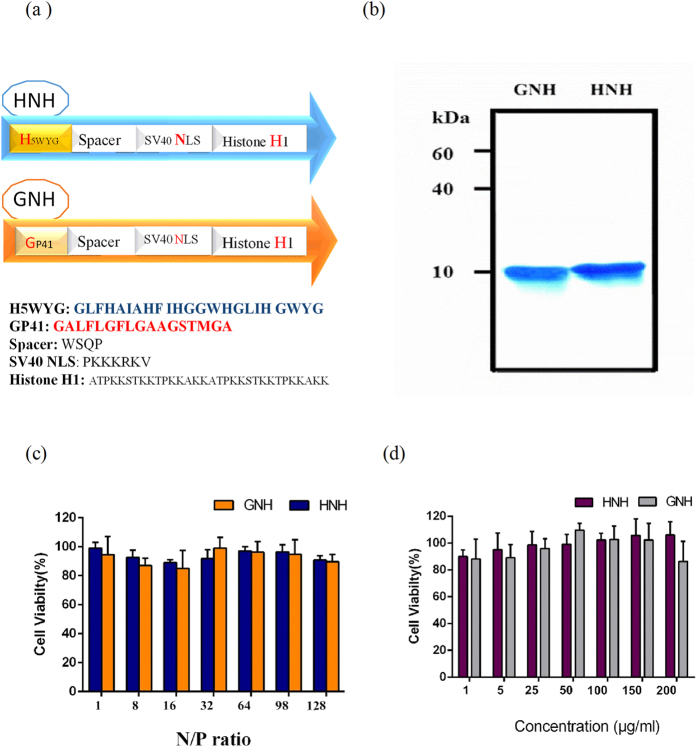
Design, production and cytotoxicity profile of HNH and GNH. (**a**) Schematic presentation of HNH and GNH peptides that are composed of multiple motif. (**b**) GNH and HNH peptides were expressed in *E. coli* BL21 (DE3) and purified using the Ni-NTA Sepharose column chromatography. The bands were visualized using CBB staining. The molecular weights of GNH and HNH are 10.1 and 10.8 kDa, respectively. Cellular viability in presence of nanoparticles and their bar nanocarrier**s**. (**c**) The HEK293T cells were incubated with HNH and GNH nanoparticles at various N/P ratios for 48 h. (**d**) The HEK293T cells were incubated with HNH and GNH nanocarriers at various concentrations for 48 h. The data were normalized to viability of untreated cell considered as 100% viable. Data are mean ± standard deviation (n = 3).

We had previously shown that H5WYG exhibited endosome escape activity when positioned at N-terminal of peptide^28^. Therefore, in this study we put all essential gene delivery motifs at the C-terminal of Gp41 and H5WYG in GNH and HNH all-in-one nanocarriers respectively (Fig. 1a) and the physiochemical properties of nanocarriers were theoretically calculated. The GNH nanocarrier showed a slightly higher hydrophobicity compared to HNH. However, both nanocarriers had the same electrical charge with almost identical PI (isoelectric pH), suitable for proper compression of plasmids (Table 1).

### Expression and characterization of nanocarriers

The coding sequences of GNH and HNH nanocarriers were cloned in pET28a expression system, which provides hexahistidine-tag sequence at both N-terminal and C-terminal of peptide-based nanocarriers (Supplementary Figure S1). Double digestion with restriction enzymes and sequencing confirmed the fidelity of each cloned sequence with the original designs. These nanocarriers were expressed in *E. coli* as inclusion bodies. Indeed, the cationic nature of these carriers are usually causing bacterial toxicity. Therefore, the carriers’ expression at 37 °C caused inclusion body formation. The inclusion bodies of carriers were successfully solubilized and purified by Ni-NTA chromatography. The SDS–PAGE and Bradford analysis showed high purity of carrier (>99%) and obtaining of a production yield of 6 and 8 mg/liter for HNH and GNH, respectively (Fig. 1b).

### The cytotoxicity profile of nanocarriers

Safety is an important factor to be considered when a gene delivery nanocarriers is designed. Furthermore, nanocarrier associated cytotoxicity can reduce the total gene delivery efficiency as well. To check the cytotoxicity profile of these nanocarriers, we examined the viability of the HEK 293 T cells, treated with either nanoparticles or bare nanocarriers of HNH and GNH using MTT assay. The result demonstrated that nanoparticles at different N/P ratio did not have any significant effect on cell viability. In fact, almost 90% of cells remained viable in presence of nanoparticles with N/P ratio of 1 to 128 within 48 h (Fig. 1c). Another word, the both nanocarriers didn’t show any significant difference in terms of cytotoxicity at examined concentrations (Fig. 1d). Therefore, it was confirmed that both carriers were safe to be considered for further examination and analysis.

### Luciferase expression assay

**Figure 2:**
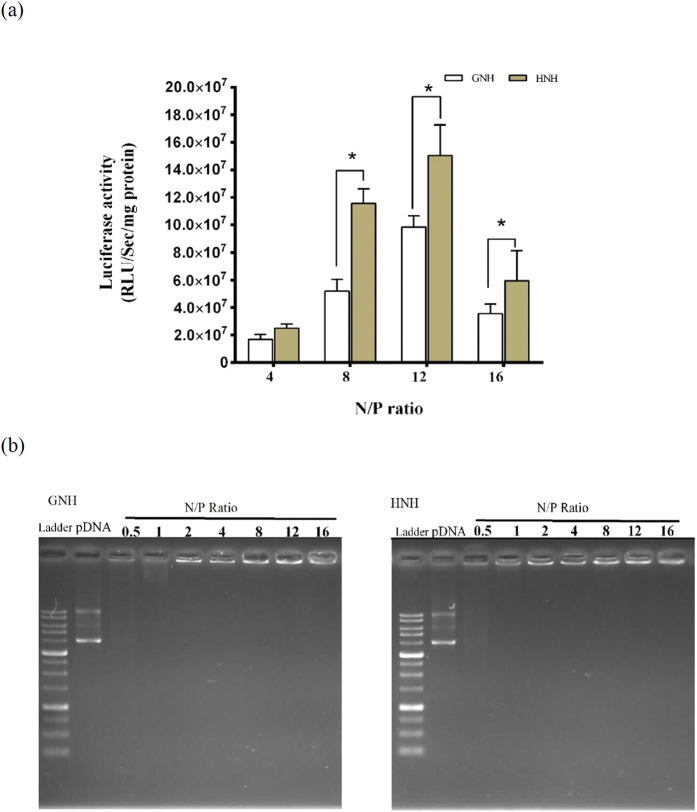
Transfection efficiency and gel retardation assay of HNH and GNH nanoparticles at various N/P ratios. (**a**) Transfection rates of the both GNH and HNH nanoparticles (Contained 1 μg pGL3 plasmid) were measured using luciferase assay in HEK293T cell’s 48 h post transfection. Total proteins (mg) were used for normalization of Relative Light Unit/Sec (RLU/Sec) values from luciferase assay. Data are mean ± standard deviation (n = 3). *p < 0.05. **(b**) Agarose gel retardation assay of GNH and HNH in function of N/P ratio of nanocarrier/pDNA complex. The nanoparticles contained 300 ng pGL3 plasmid and similar amount of this plasmid was used as control pDNA. Ladder: 1 kb DNA ladder.

We examined the endosomal release activity of H5WYG and Gp41 peptides using the designed HNH and GNH carriers. Therefore, pGL3 plasmid was transfected with these carriers at N/P ratios of 4 to 16. The result shows that both nanocarriers facilitated the delivery of pGL3 to HEK293T cell nucleus, which led to luciferase gene expression (Fig. 2a). In addition, both nanoparticles demonstrated a significant increase of transfection rate from N/P ratio of 4 to 12 (P ≤ 0.05). However, the transfection rate of nanoparticles suddenly dropped at N/P ratio of 16. Surprisingly, the HNH exhibited a higher transfection efficiency than GNH nanocarriers at examined N/P ratios. For instance, at N/P ratio of 8, the luciferase activity was 2 fold higher for HNH carrier compare to GNH carrier at the same N/P ratio. Nevertheless, additional studies were needed to further confirm these observations and to evaluate the other possible factors involved in the superior activity of H5WYG flanked carrier.

### DNA binding ability of nanocarriers

The plasmid DNA molecule is strongly repulsed by cell membrane due to its polyanionic nature; therefore, charge neutralization step is necessary for an efficient intracellular gene delivery. Based on theoretical calculation, at N/P ratio of 1 the nanoparticles are formed with the minimum amount of nanocarriers, sufficient to neutralize the plasmids. As shown in Fig. 2b, both HNH and GNH efficiently bound to plasmids and retard their electrophoretic movements. Indeed, the presence of illuminating plasmid in each well is an indicator of gel retardation and neutralization of plasmid charges Both carriers began to neutralize the negative charges of plasmids at N/P ratio of 0.5 and it became completely neutralized when carrier concentration was increased to N/P ratio of 1. At higher N/P ratios, nanoparticles completely remained in the wells, so that SDS treatment of these nanoparticles led to release of their plasmid contents (Supplementary Figure S2). It is noteworthy to mention that the intensity of retarded plasmid was weak at N/P ratio of 16, presumably through less ethidium bromide accessibility. The gel retardation analysis showed that, both nanocarriers similarly neutralized the negative charges of plasmid and binding force between carrier and plasmid would not act as a differential factor in gene delivery rate of these carriers.

### Serum Stability

**Figure 3:**
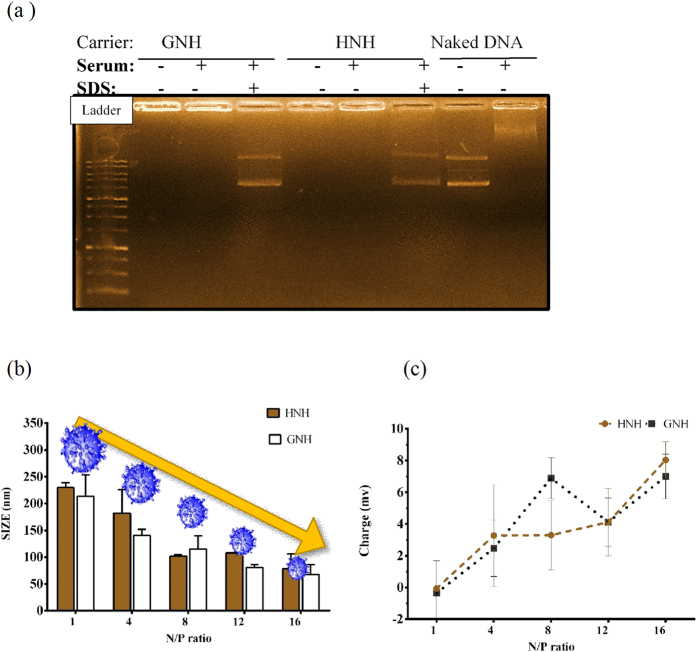
Serum stability and DLS analysis of GNH and HNH nanoparticles. (**a**) Serum stability of peptide based nanoparticles. The GNH and HNH nanoparticles at N/P ratio of 8 were incubated with serum at a final concentration of 10% for 30 min. serum nuclease activity was stop by treatment of nanoparticle at 65 °C for 20 min and then 2 mM SDS treatment released pDNA from nanoparticles. Untreated Nanoparticle, Naked DNA and serum treated DNA were used as control. DLS analysis of charge and size of nanoparticles. (**b**) GNH and HNH nanoparticles size analysis, (**c**) GNH and HNH nanoparticles surface charge analysis. GNH and HNH carriers were complexed with pgl3 plasmid at different N/P ratio. The results are reported as ± standard deviation (n = 3).

DNA protection from serum nucleases is an essential requirement for an efficient gene delivery system. The stability of nanoparticles and their nuclease protection capability were visually examined by gel mobility assay at N/P ratio of 8. The result demonstrated that these nanoparticles remained stable in the presence of serum and were not dissociated into their components. While both HNH and GNH carriers efficiently covered and protected plasmids from serum nuclease break down, the naked plasmids on the other hand, were degraded in presence of serum (Fig. 3a). The higher molecular weight of plasmid upon treatment with serum is probably come from macromolecular assembly of serum proteins and digested plasmid DNA, which bring about with lower gel migration in Agarose electrophoresis^43^. The SDS treatment of our complex showed that the released plasmids had remained intact and their integrity was preserved in the complex consists of these carriers. This result suggested that both nanoparticles are capable of protecting the DNA content from cytoplasmic nucleases and most likely are stable under *in vivo* conditions^44^.

### Nanoparticle’s charge and size analysis

The physicochemical properties of nanoparticles can affect the uptake rate, intracellular fate and cell entry mechanism^45^. Therefore, the size and the surface charges of HNH and GNH nanoparticles were characterized at different N/P ratios The DLS analysis showed that both nanocarriers condensed plasmids into positively charged particles with less than 180 nm in size. These results point out that histone H1 as a condensing motif worked appropriately in both nanocarriers. As shown in Fig. 3b, a statistically significant compactness and an increase in surface charges of nanoparticles were occurred with increasing N/P ratios for both NHN and GNH nanocarriers. The nanoparticles with N/P ratios between 8–20, which showed a size range between 60–125 nm and a zeta potential between 4–10 mV, were suitable to interact with cell membrane and initiate cellular uptake via the endocytosis pathway^46^ (Fig. 3c). However, the HNH and GNH nanoparticles did not show a statistically significant difference in terms of size and zeta potential (p > 0.05).

The particle size and morphology of HNH and GNH nanoparticles at N/P ratio of 10 were also characterized via transmission electron microscopy. TEM indicates that both HNH and GNH in complex with plasmid can form condensed semi-spherical particles with a size around 100 nm for both, which is in concurrence with DLS results (Supplementary Figure S3a). Atomic Force Microscopy also revealed surface topology of bare HNH and GNH nanocarriers (Supplementary Figure S3b).

### Cell entry rate

**Figure 4:**
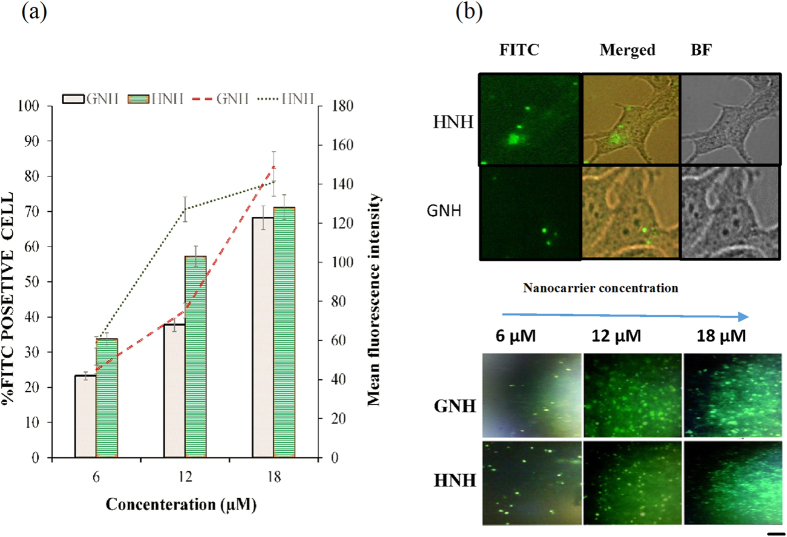
Cellular uptake of FITC-labeled HNH and GNH nanocarriers. (**a**) The percentage of FITC positive cells (bar) and the mean fluorescence intensity (line) were investigated 8 h after of incubation of HEK293T cells with these carriers at different concentrations. (**b**) Uptake of these carriers in live cells were studied using Fluorescence microscopy. Cells were visualized at 40X, and 4X magnifications. Scale bar: 20 μm.

To understand the mechanism behind the observed differences between HNH and GNH transfection efficacy, we analyzed the cellular uptake of the FITC labeled nanocarriers. As shown in Fig. 4a, the uptake rate of nanocarriers was increased as a function of their concentration. The main fluorescence intensity index demonstrated a superior uptake per cell for HNH nanocarrier. However, at higher concentration, both nanocarriers showed a similar uptake rate and entered into more than 70% of the cells. The fluorescence microscopy also confirmed the intracellular localization of FITC-labeled nanocarriers (Fig. 4b).

### Hemolysis assay

**Figure 5:**
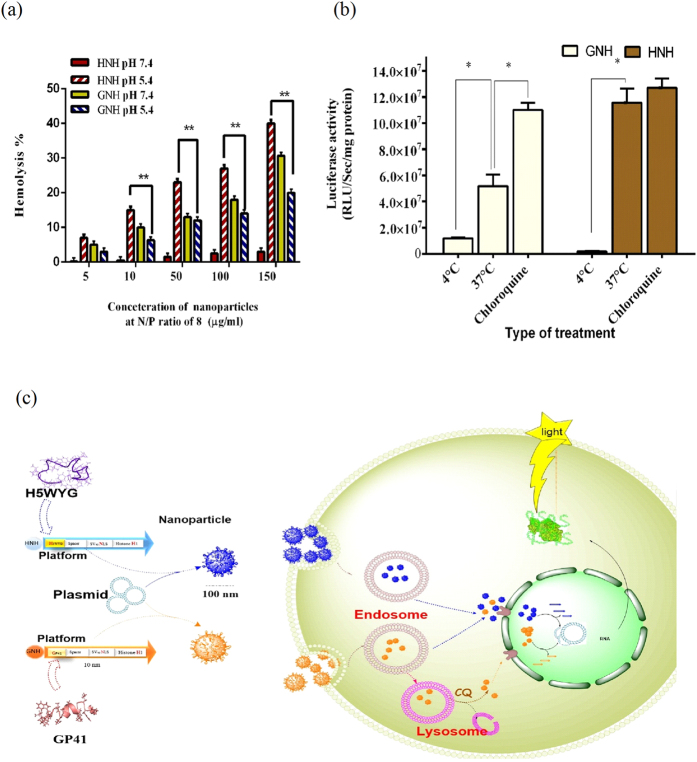
Hemolysis assay, cell entry mechanism and intracellular fate of GNH and HNH Nanoparticles. (**a**) Nanoparticles (Contained 0.5–15 μg pGL3 plasmid) at N/P ratio of 8, in the acetate buffer (pH 5.4) and phosphate saline buffer, PBS (pH 7.4). Triton-x100 and buffers were used as positive and negative control, respectively. (**b**) The Nanoparticles (Contained 1 μg pGL3 plasmid) at NP ratio 8 were transfected in HEK293T at different temperature (4 °C and 37 °C) and in the presence of chloroquine (100 μM) at 37 °C. Luciferase activity was monitored 48 h later and reported as a function of total protein. The date was reported as mean ± SD. (n = 3). *p < 0.05,**p < 0.001. (**c**) Schematic diagram of intracellular trafficking of GNH and HNH Nanoparticles.

The Endosomal release capability of nanoparticle usually affects the efficacy of gene delivery. Red blood cells were used as *ex-vivo* model for endosome. A hemolysis assay was carried out to investigate and compare the membrane release activity of HNH and GNH nanoparticles at physiological pH (7.4) and endosomal pH (5.4). As shown in Fig. 5a, both of nanoparticles were able to lyse red blood cells at pH 5.4 at different concentrations. However, the HNH showed a statistically significant higher activity than GNH at this pH (P ≤ 0.01). GNH nanoparticles, on the other hand, demonstrated the hemolytic activity between 6 to 30% as a function of nanoparticles concentration at pH 7.4, while the HNH nanoparticles lost their activities at this pH. The pH–sensitive characteristic of HNH carrier provides a hemo-compatible property for future *in vivo* application. These findings alongside with cell entry rate results, suggest that efficacy of carriers can be controlled by their endosomal release motifs.

### Cellular uptake mechanism and intracellular fate of nanoparticles

The mode of cell entry usually affects the intracellular fate and overall delivery rate of nanoparticles. In this study; the cell entry mechanism of GNH and HNH nanoparticles were investigated by transfection of luciferase gene at 4 °C for 4 h followed by incubation at 37 °C for 44 h^47^. Low temperature (4 °C) diminished the transfection rate of both nanoparticles. As shown in Fig. 5b, the transfection efficacy of both HNH and GNH was reduced significantly at low temperature. Endocytosis was indeed the main uptake pathway for both nanoparticles since it was significantly inactivated at low temperature^45^,^48^. In another attempt, we examined endosomal escape potency of internalized nanoparticles using chloroquine, which disrupts endosome via an osmotic effect^49^. Therefore, transfection was performed in the presence of chloroquine to determine the content of entrapped nanoparticles inside the endosome. The transfection rate of GNH nanoparticle was increased two-fold in the presence of chloroquine. The transfection rate of HNH nanoparticle, however, showed a slightly increase under the same condition. Overall, the results of this study, indicated that both of nanoparticles were entered into the cell by an energy-dependent mechanism like endocytosis and escaped from endosome with different efficiency (Fig. 5c). Based above-mentioned documents, the luciferase activity could mirror the superior endosome release activity of HNH, which is equipped with H5WYG motif, compared to GNH, which is equipped with Gp41motif.

## Structure analysis

### Modelling 3D structure of carriers

**Figure 6:**
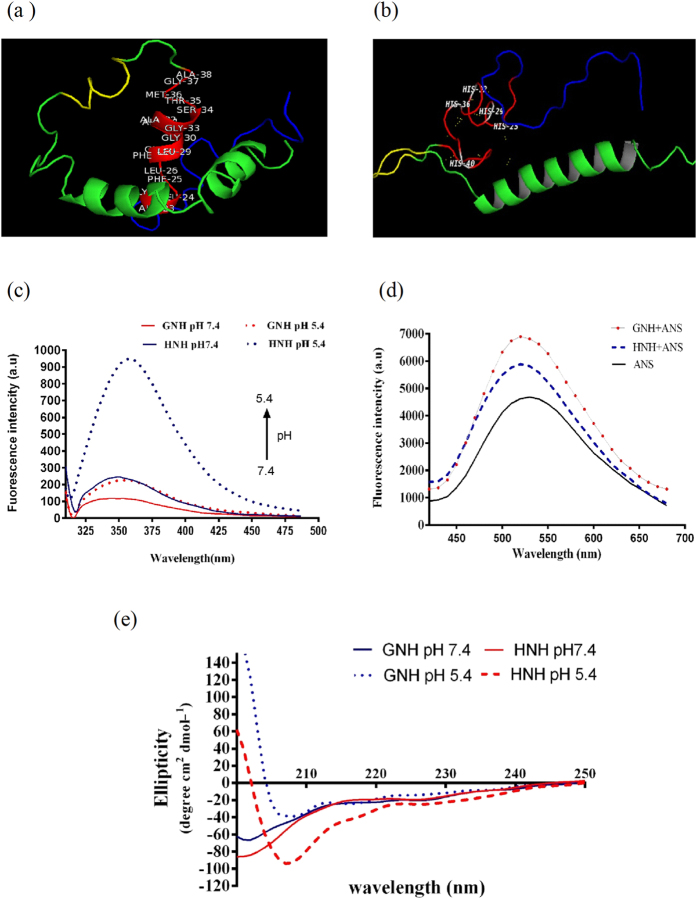
Structural analysis of nanocarriers. The three-dimensional structure of nanocarriers were predicted by I-TASSER server. Different motif of nanocarriers were depicted with multiple colors by PYMOL software. Red color: endosome releasing motif, Green: DNA condensing motif, Yellow: nuclear localization signal and blue color: Ni –NTA binding domain. (**a**) Presentation of HNH nanocarrier, the position of five histidine residues have been shown on H5WYG motif of HNH nanocarrier. (**b**) Presentation of GNH nanocarrier, in which the sequence of GP41 motif has been shown on its structure. Intrinsic and extrinsic fluorescence emission of GNH and HNH carrier. **(c)** Fluorescence spectra of GNH and HNH in the acetate buffer (pH 5.4) and phosphate buffers saline (pH 7.4) were recorded under 295 nm excitation wavelength. (**d)** Extrinsic fluorescence of these nano carriers in mixture with ANS (1:30 ratio) were recorded under 450 nm excitation. (**e**) Influence of the pH shift on the far UV CD spectrum of nanocarriers. The CD spectra GNH and HNH nanocarriers at concentrations of 0.2 mg/ml in acetate buffer pH 5.4 and PBS pH 7.4.

I-TASSER server was used to model the secondary and tertiary structure of carriers. As shown in Fig. 6a, the H5WYG motif, demonstrated a random coiled structure that was consistent with its ancestor motif at physiological conditions^29^. The Gp41 motif showed a helix structure (Fig. 6b). To confirm these findings, we further analyzed the structure of nanocarriers with fluorescence spectroscopy and circular dichroism spectropolarimetry. The lack of secondary structure of SV40 NLS motif, provides a suitable condition for importin machinery to recognize its binding site on the nanocarriers^50^.

### The intrinsic and extrinsic fluorescence of nanocarriers

The pH dependency of nanocarriers structures were investigated by measuring the intrinsic fluorescence. Peptide with endosomal escape property must remain active or configured at suitable form at low pH. The spacer motif of the carrier has a Trp residue that acts as an intrinsic fluorescence probe. The fluorescence analysis indicated that the pH reduction did not considerably affect the structure and the polarity of environment surrounded the Trp residue of GNH nanocarriers. In contrast, the HNH nanocarrier showed a drastic fluorescence enhancement at low pH, reflecting a significant change of the Trp surrounding environment (Fig. 6c). This result suggested a conformational change in the nanocarrier at acidic pH of endosome.

Hydrophobic interactions have shown an important role for peptide membrane interaction^51^,^52^. Therefore, we investigated the hydrophobic patch of carriers with ANS as a sensitive probe. As it is shown, both carriers increased the fluorescence intensity of the ANS probe (Fig. 6d). Furthermore, GNH showed a higher fluorescence intensity compared to HNH, which indicate that the GNH has more hydrophobic pockets for ANS binding.

### Secondary structure of nanocarriers

The Far-UV circular dichroism analysis were performed to evaluate the influence of pH on the secondary structure of carriers. The GNH demonstrated a CD spectrum with a single minimum at 198 nm, which indicate a random coiled conformation. Further, this carrier revealed two typical maximums at 200 nm and 190 nm as well as a minimum at 207 nm at 5.4 pH, suggesting a decrease of non-ordered structure. However, at low pH the CD spectrum in the deterministic region (205–230 nm) showed a similar manner to neutral pH. The CD spectrum of HNH peptide at neutral pH showed a single minimum at 195 nm, which is an indicative of non-structured conformation. Surprisingly, this nanocarrier at low pH showed a CD spectrum with a maximum at 200 nm associated with a minimum at 207 nm, the signature of helical conformation (Fig. 6e) indicated that secondary structure of HNH carrier drastically changes at low pH compared to GNH carrier.

## Discussion

Endosome is the main bottleneck in intracellular gene delivery path. During the last few decades, several de novo designed or biologically derived endosomal release peptides have been reported^30^,^37^,^53^,^54^. However, the complicated mechanisms underlying endosome release and the inability of these peptides to show their actual activities, makes it more difficult to have a preference for selecting the most efficient and the most effective ones. Most recently, several groups have used genetic engineering approach to develop a single-chain peptide based carrier, which is able to overcome all intracellular barriers solely. Fusogenic peptides of HIV GP41 and influenza–derived H5WYG are two peptides with endosome release ability, which have been used to enhance the efficacy of these carriers in gene delivery.

In this study, we designed two peptide based nanocarriers with four essential motifs assembled in a single chain of peptide to check the efficacy of these motifs for gene delivery and to investigate their mechanism of actions (Fig. 1a). The first embedded motif was comprised of two repeats of 16 mer Histone H1 (ATPKKSTKKTPKKAKK), which packs DNA in a particle with electrostatic binding forces^28^. The second functional motif was the NLS of simian virus 40 (SV40) large T antigen required for nuclear localization^55^,^56^. The third was an UV-active spacer motif contained a Tryptophan residue, which acts as a sensitive probe for spectroscopy analysis. The fourth functional motif was either HIV GP41 or H5WYG fusogenic peptides that are individually flanked at N-terminal position of the spacer motif in GNH and HNH carriers, respectively. The quantity (8 mg/L of culture) of our purified carriers confirmed the ability of *E. coli* bacteria in biosynthesis of the long poly-cationic sequences (Fig. 1b). Electrostatic interactions between plasmid and carriers allowed us to prepare the identical nanoparticles in terms of their component’s stoichiometry. As shown in Fig. 1c the MTT assay confirmed the safety of both carriers, which is a distinct advantage in using biomimetic carriers. Transfection of nanoparticles containing the luciferase gene provides a simple, robust and fast technique to detect the rate of intracellular plasmid delivery and gene expression. The transfection results clearly indicate a N/P ratio-dependent delivery rate for both HNH and GNH (Fig. 2a). The superior transfection efficiency of HNH carrier suggests, higher endosomal release capacity for H5WYG motif.

To exclude the possibility of other nanoparticles properties that might affect the transfection rate, a series of *in vitro* analysis were carried out to investigate different aspects of their behaviors. The gel retardation analysis showed that, both nanocarriers neutralize the negative charges of plasmid at N/P ratio of 1, being consistent with the theoretical N/P ratio (Fig. 2b). The result also confirmed that the electrostatic interactions are the main particle stabilizer forces, which remain stable under physiological conditions (150 mM NaCl and pH 7.4). Therefore, the binding force between carriers and plasmids could not be considered a differential factor in determining the gene delivery rate for these nanocarriers.

Degradation of DNA by nucleases present in serum, endosome and cytoplasm can effect and reduce the plasmid delivery rate^44^. The results of serum stability test indicated that both HNH and GNH carriers shielded the plasmids from serum nuclease activities thus prevented their degradation and clearance by serum components (Fig. 3a). Thus, the presence of nucleases does not reduce the transfection rate of GNH nanoparticles.

The size of particle which in turn refers to the compactness of DNA/carrier complex, can affect the cell entry pathway and the rate of plasmid release from complex^17^. According to DLS results, both nanoparticles showed a size around 100 nm (Fig. 3b), where usually nanoparticle uptake happens through a clathrin mediated pathway^45^,^57^. Furthermore, a similar condensation rate for both carriers was confirmed by particle size analysis. Due to inverse relationship between particle compactness and gene expression^17^, the extreme compactness of nanoparticles at N/P ratio of 16 decreased the release rate of plasmid into the nucleus. However, the transfection rates of HNH and GNH were significantly different at N/P ratio of 16. Hence, these results reveal a similar plasmid release rate per particle for both carriers.

Particle surface charge usually affects the solubility, and the electrostatic interaction with cell membrane, which in turn initiates the endocytosis^58^. Zeta potential analysis showed that both nanoparticles had similar partial positive charges at transfected N/P ratios (Fig. 3c). Therefore, it seems that the particle charge was not a determinant factor in transfection rate.

It is difficult to predict the uptake rate of carriers based on their primary structures. The FACS results confirmed that the FITC-labeled carriers, entered the cells with a first-order kinetic rate, without any statistically significant differences (Fig. 4a). Thus far, the results of GNH and HNH *in vitro* analyses were consistent with our hypothesis. As expected, these particles showed a significant difference in terms of hemolytic ability (Fig. 5a). Another word, the hemolysis assay confirmed the activation of membrane lytic ability of HNH nanoparticles at low pH, where the GNH showed an activity decrement. On the other hand, the transfection results confirmed that the endocytosis is the main cell entry pathway for both carriers.

Furthermore, the chloroquine treatment triggered the release of a large amount of endosome-entrapped GNH nanoparticles and increased the transfection rate for GNH carrier, while the transfection rate of HNH didn’t show any significant increment under the same condition (Fig. 5b,c). This finding confirmed a significantly higher endosome release activity for HNH carrier equipped with H5WYG compared to GNH carrier, which was consistent with hemolysis assay results^25^.

Overall, carrier associated parameters including particles size and charge, DNA binding, nuclease protection and cell entry rate didn’t play any role in the activities of GNH and HNH carriers. Meanwhile, the endosomal release capability, consistent with luciferase assay, completely controlled the efficacy of carriers. Consequently, these results shed light on the precise origin of superior transfection rate of HNH carrier and confirmed the ability of our designed all-in-one carriers to provide a similar condition for evaluating the endosomal release motif independent of other intra carrier interactions.

These surprising results reinvigorated us to elucidate the mechanism underlying the observed endosomal release activities. We evaluated the structure of HNH and GNH carriers with theoretical modeling and sensitive experimental analysis. The projected 3D model confirmed that the five histidine residues are spatially in close proximity of H5WYG endosome release motif (Fig. 6b). The ionization state of these residues is changed during endosomal escape, which might lead to their pH dependent structure. GP41 on the other hand, showed an amphipathic helical structure (Fig. 6a). Fluorescence analysis of Trp residues confirmed a pH dependent structural change in HNH carrier (Fig. 6c). However, the results of extrinsic fluorescence analysis with ANS confirmed that GNH carrier has a higher content of hydrophobic patches compared to HNH carrier (Fig. 6d). This result suggests that hydrophobicity may be the main reason for interaction of GNH with membrane. Therefore, the GP41 motif may work upon uptake of nanoparticle at natural pH. In contrast, circular dichroism analysis confirmed that the HNH carrier forms a helical structure at low pH (Fig. 6e) that is suitable for the particle’s fusion with endosomal membrane and its subsequent release in the cytoplasm. In conclusion, we have studied the ability of our designed peptide based carriers to evaluate and compare H5WYG and GP41 endosomal escaping motifs in cellular environment. The results obtained from this study suggest that, H5WYG provides a better tool to design an effective and efficient gene delivery system, compare to Gp41. Furthermore, we attempted to explain the differences we observed in endosomal release abilities of these motifs. This study suggested that, a shift in secondary structure of H5WYG at low pH resulted in its sharp release from endosome. Altogether, Nano-biomimetic carriers provide a suitable condition for mechanistic comparison of endosomal release motifs and can be used for screening different functional motifs to maximize the efficacy of gene delivery systems.

## Materials and Methods

### Plasmid construction and cloning of peptide based carriers

The genes encoding HNH and GNH carriers were designed, expression optimized and synthesized by Eurofins MWG Operon (Ebersberg, *Germany*). The DNA sequences were double digested with *Nd*eI and *Xh*oI restriction enzymes and ligated in pET28a expression vector (Merck KGaA, Darmstadt, Germany), which provide hexahistidine-tag sequence at both N-terminal and C-terminal. To verify the fidelity of the designed sequences, the plasmid containing HNH and GNH were sequenced using an automatic sequencer (MWG) by T7 promoter universal primer.

### Expression and purification

The expression vectors were transformed into *Escherichia coli* bl21 (DE3) pLysS (Novagen) and the E. coli was cultured in LB medium containing kanamycin (100 μg/ml) at 37 °C overnight. One ml of pre-cultured bacteria was used to inoculate 2-XY medium (250 ml) and incubated at 37 °C while shaking at 220 rpm. At optical density (OD_600_) of 1.5, peptide expression was induced by addition of IPTG to a final concentration of 0.4 mM at 37 °C for 4 h. The bacterial cells were harvested by centrifugation at 5000 RPM for 15 min.

The cell pellets were resuspended in lysis buffer [20 mM Tris-HCl, 500 mM NaCl, 8 M urea and 5 mM imidazole; (pH 12)] and sonicated at 20-s bursts for 10 times. The lysate was centrifuged at 13000 rpm for 30 min and the supernatant was loaded on a Ni-NTA column chromatography. The impurities were removed by 15 ml of wash buffer [20 mM Tris-HCl, 1000 mM NaCl and 20 mM imidazole; (pH 8)] and the peptide-based carriers were then eluted by cold elution buffer [20 mM Tris-HCl, 150 mM NaCl and 250 mM imidazole; (pH 8)]. The purity and relative concentration of the purified carriers were analyzed by 17.5% sodium dodecyl sulfate/polyacrylamide gel electrophoresis (SDS-PAGE) and Coomassie blue staining. Physicochemical properties of these carriers were theoretically calculated by ExPASy’s tools^59^.

### Preparation of nanoparticles

Nanoparticles were produced by complexation of nanocarriers and pGL3 plasmids at different N/P Ratio. The N/P ratio refers to the molar ratio of the amine groups of the carriers to the phosphate groups of Plasmid. Theoretical N/P ratios were calculated according to equation (1).


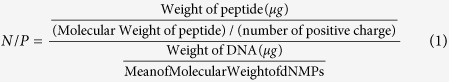


where the charges of Lys and Arg residues were considered +1, while His residue as zero. For instance, HNH and GNH nanoparticles at N/ratio of 1 were prepared by mixing 1 μg of pGL3 plasmid with 1.5 μg of HNH carrier and 1.4 μg of GNH carrier, respectively. After that, the complexes immediately were mixed and incubated at 25 °C for 45 min.

### Cell viability assay

The potential cytotoxicity of nanocarriers were evaluated with MTT assay (Sigma, USA). The Hek293T (7 × 10^3^) cells were seeded in 96 wells plate and incubated for 24 h at 37 °C in a humidified incubator with 5% CO_2_ atmosphere. The medium was replaced with the serum-free DMEM medium supplemented with either nanoparticles (containing 0.2 μg plasmid) at different N/P ratios or various concentrations of bare nanocarriers. After 4 h, FBS was added to the cells at a final concentration of 10% (v/v). To assay the cell viability, 10 μl of MTT reagent (5 mg/ml) was added to each well and allowed to react at 37 °C for 4 h. To dissolve the formazan crystals, 100 μl of DMSO was added into each well and the absorbance was measured using a micro plate reader (ELx800, Biotek, USA) at 570 nm. The absorbance of untreated cell was considered as 100% viable. The results are reported as mean± SD (n = 3).

### Transfection and Luciferase assay

The HEK 293 T cells were used as a model to evaluate the transfection efficiently of each nanocarrier. Briefly, 4 × 10^4^ cells were seeded in 24 well tissue culture plate in a humidified incubator at 37 °C under 5% CO_2_ atmosphere, two days before transfection. The nanoparticles (containing 1 μg PGL3 in complex with HNH and GNH nanocarriers) were prepared at different N/P ratios and adjusted to 100 μl with serum-free DMEM medium. At transfection time, the media was completely removed, and nanoparticles were added drop-wise to each well. The DMEM medium supplemented with 10% FBS and 1% (v/v) penicillin–streptomycin was added 4 h later. In this study, firefly luciferase gene expression encoded by PGL3 plasmid was measured according to previously reported procedure^60^. Briefly, 48 h post transfection, the cells were lysed by cell lysis buffer (Promega, USA). The luciferase activity was measured in the presence of firefly luciferase substrates [2 mM Luciferin, 4 mM ATP and 100 mM MgSO_4_ 100 mM (pH 7.8)] with a luminometer (Berthold detection systems, GmbH). The results were normalized with respect to their protein concentrations. The data are indicated as the relative light unit (RLU/sec)/mg of total proteins and presented as mean ± SD (n = 3).

### Characterization of Nanoparticles

#### Gel retardation

The DNA binding abilities of HNH and GNH carriers were examined by Gel Retardation assay. Nanoparticles contained 0.3 μg plasmid at different N/P ratios were loaded on agarose gel (1%) and left to run at 80 V for 1 h. The mobility of plasmid was visualized by Ethidium bromide staining and UV illumination. GNH and HNH nanoparticle contained 0.3 μg pGL3 plasmid at N/P ratio of 4, 8 and 12 were prepared in two groups. The one of them was directly electrophoresed using agarose gel. Another group was incubated with SDS 10% and then was electrophoresed.

#### Particle size and charge analysis

Size and zeta potential measurements of nanoparticles were performed using Dynamic Light Scattering (DLS) by a Malvern Nano ZS instrument and DTS software (Malvern Instruments, UK). Briefly, the complexes of plasmid and nanocarriers were prepared at different N/P ratios, as mentioned above. The volume of each sample was adjusted up to 1 ml by cold PBS. The results were analyzed by Zetasizer software (V6. 12) and presented as a mean of three independent batches for each N/P ratio.

#### Hemolysis assay

The protocol used in this study conformed to the standards set by the Declaration of Helsinki which is approved by ethic committee of Tarbiat Modares University. Human blood was collected from healthy volunteers with providing of written consent, into heparinized vacutainers under aseptic conditions. The human whole blood cells were centrifuged at 5000 rpm for 5 min. The supernatants were discarded, and the Red blood cells (RBC) were washed three times with PBS. The 108 RBCs were resuspended in 1 ml of PBS buffer pH7.4 or acetate buffer pH 5.4 supplemented with 0.9% NaCl. Nanoparticles at N/P ratio of 8 with final concentration between 5 to 150 μg/ml were added to the cell suspensions and incubated at 37 °C with mild shaking for 1 h. The tubes were centrifuged for 5 min at 5000 rpm, and the absorbance of the supernatant was measured at 541 nm. Untreated RBCs (pH 7.4 and 5.4) were used as negative controls, while triton X-100 (1%) was used a positive control. The percentage of hemolysis was calculated according to equation (2). Each treatment was repeated three times and the t-tests (P < 0.05) was used to evaluate the statistical significance. The results are reported as mean± SD.


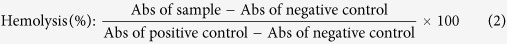


### Cellular Uptake and intracellular analysis

#### Cellular uptake of nanocarriers

HEK293T cells (4 × 10^5^) were seeded in a 6 well plate in DMEM medium supplemented with 10% (v/v) FBS and 1% (v/v) penicillin–streptomycin prior to the experiments for 24 h. The FITC-labeled nanocarriers were added at different concentrations to the cells in presence of complete DMEM medium. After 3 h incubation at 37 °C, the cells were washed twice with PBS. To determine the cellular localization of these carriers, the cells were visualized using a Nikon Eclipse TE2000-S fluorescence microscope (Nikon Instruments, Japan), prior to flow cytometry analysis. The flow cytometry were performed to assess the cellular uptake of each carrier. Briefly, the cells were detached using 0.025% trypsin, and centrifuged at 1200 RPM for 5 min. The pellets were suspended in 0.5 mL PBS, kept in ice and analyzed using a FACS Calibur flow cytometer (FACS, Becton-Dickinson, USA). The result was analyzed by flowing software (Turku University, Finland).

#### Uptake pathway and endosome release analysis

Transfection was carried out at a low temperature to examine the endocytosis dependency of HNH and GNH nanoparticles uptake pathways. The HEK 293 T cells were seeded at density of 4 × 10^4^ cells per well for two days. When the cells became 70% confluent, the first group were incubated at 4 °C for 1 h prior to transfection. They were then incubated at 4 °C with nanoparticles at N/P ratio of 8 (according to transfection result) for another 4 h. In order to eliminate free nanoparticles, cells were rinsed with PBS before adding fresh complete DMEM medium to each well. The cells were incubated at 37 °C for additional 44 h. To examine the endosome releasing activity of each nanocarrier in a real environment, the second group of cells were transfected in presence of chloroquine (CQ) (Sigma-Aldrich, USA) at a final concentration of 0.1 mM at 37 °C. The luciferase assay was performed as mentioned above 48 h later. The relative light units/Sec (RLU/Sec) was normalized for each sample with respect to their protein concentrations. The experiment was performed in triplicate, and the result was analyzed using student’s test analysis.

## Additional Information

**How to cite this article**: Alipour, M. *et al*. Nano-biomimetic carriers are implicated in mechanistic evaluation of intracellular gene delivery. *Sci. Rep.* **7**, 41507; doi: 10.1038/srep41507 (2017).

**Publisher's note:** Springer Nature remains neutral with regard to jurisdictional claims in published maps and institutional affiliations.

## Supplementary Material

Supplementary Information

## Figures and Tables

**Table 1:** Physicochemical properties of peptides that calculated theoretically by EXPASSY server.

Name	MW (Da)	PI*	Hydrophobicity	Charge
HNH	10995.7	11.19	29.17%	+23
GNH	10083.8	11.56	30.43%	+23

*Isoelectric point.

## References

[b1] Davidson B. L. & McCray P. B. Current prospects for RNA interference-based therapies. Nature Reviews Genetics 12, 329–340, doi: 10.1038/nrg2968 (2011). PMC7097665 21499294

[b2] Fonseca S. B., Pereira M. P. & Kelley S. O. Recent advances in the use of cell-penetrating peptides for medical and biological applications. Advanced Drug Delivery Reviews 61, 953–964, doi: 10.1016/j.addr.2009.06.001 (2009). 19538995

[b3] Kim D. H. & Rossi J. J. Strategies for silencing human disease using RNA interference. Nature reviews. Genetics 8, 173–184, doi: 10.1038/nrg2006 (2007). 17304245

[b4] Mastrobattista E., van der Aa M. A., Hennink W. E. & Crommelin D. J. Artificial viruses: a nanotechnological approach to gene delivery. Nature reviews. Drug discovery 5, 115–121, doi: 10.1038/nrd1960 (2006). 16521330

[b5] Li S. D. & Huang L. Gene therapy progress and prospects: Non-viral gene therapy by systemic delivery. Gene Therapy 13, 1313–1319, doi: 10.1038/sj.gt.3302838 (2006). 16953249

[b6] Wang F., Hu K. & Cheng Y. Structure-activity relationship of dendrimers engineered with twenty common amino acids in gene delivery. Acta Biomaterialia 29, 94–102, doi: 10.1016/j.actbio.2015.10.034 (2016). 26525113

[b7] Tonelli F. M. P. . Functionalized nanomaterials: Are they effective to perform gene delivery to difficult-to-transfect cells with no cytotoxicity? Nanoscale 7, 18036–18043, doi: 10.1039/c5nr04173b (2015). 26486874

[b8] Qiu C. . Systemic delivery of siRNA by hyaluronan-functionalized calcium phosphate nanoparticles for tumor-targeted therapy. Nanoscale 8, 13033–13044, doi: 10.1039/c6nr04034a (2016). 27314204

[b9] Akrami M. . Tuning the anticancer activity of a novel pro-apoptotic peptide using gold nanoparticle platforms. Scientific Reports 6, doi: 10.1038/srep31030 (2016). PMC4977985 27491007

[b10] Cheraghi R., Nazari M., Alipour M., Majidi A. & Hosseinkhani S. Development of a Targeted anti-HER2 scFv Chimeric Peptide for Gene Delivery into HER2-Positive Breast Cancer Cells. International Journal of Pharmaceutics 515, 632–643, doi: 10.1016/j.ijpharm.2016.11.008 (2016). 27825868

[b11] Chinnasamy D. . Gene therapy using genetically modified lymphocytes targeting VEGFR-2 inhibits the growth of vascularized syngenic tumors in mice. Journal of Clinical Investigation 120, 3953–3968, doi: 10.1172/JCI43490 (2010). 20978347 PMC2964987

[b12] Liu S. . Gene and doxorubicin co-delivery system for targeting therapy of glioma. Biomaterials 33, 4907–4916, doi: 10.1016/j.biomaterials.2012.03.031 (2012). 22484049

[b13] Camp E. R. . Transferrin receptor targeting nanomedicine delivering wild-type p53 gene sensitizes pancreatic cancer to gemcitabine therapy. Cancer gene therapy 20, 222–228, doi: 10.1038/cgt.2013.9 (2013). 23470564 PMC3746039

[b14] Cutrera J. . Discovery of a linear peptide for improving tumor targeting of gene products and treatment of distal tumors by IL-12 gene therapy. Molecular Therapy 19, 1468–1477, doi: 10.1038/mt.2011.38 (2011). 21386825 PMC3149172

[b15] Kim H. A., Nam K. & Kim S. W. Tumor targeting RGD conjugated bio-reducible polymer for VEGF siRNA expressing plasmid delivery. Biomaterials 35, 7543–7552, doi: 10.1016/j.biomaterials.2014.05.021 (2014). 24894645 PMC4090046

[b16] Urnauer S. . Sequence-defined cMET/HGFR-targeted Polymers as Gene Delivery Vehicles for the Theranostic Sodium Iodide Symporter (NIS) Gene. Molecular Therapy, doi: 10.1038/mt.2016.95 (2016). PMC5023389 27157666

[b17] Pack D. W., Hoffman A. S., Pun S. & Stayton P. S. Design and development of polymers for gene delivery. Nature Reviews Drug Discovery 1, 581–593, doi: 10.1038/nrd939 (2005). 16052241

[b18] Blanco E., Shen H. & Ferrari M. Principles of nanoparticle design for overcoming biological barriers to drug delivery. Nat Biotechnol 33, 941–951, doi: 10.1038/nbt.3330 (2015). 26348965 PMC4978509

[b19] Gilleron J. . Image-based analysis of lipid nanoparticle-mediated siRNA delivery, intracellular trafficking and endosomal escape. Nature Biotechnology 31, 638–646, doi: 10.1038/nbt.2612 (2013). 23792630

[b20] Martens T. F., Remaut K., Demeester J., De Smedt S. C. & Braeckmans K. Intracellular delivery of nanomaterials: How to catch endosomal escape in the act. Nano Today 9, 344–364, doi: 10.1016/j.nantod.2014.04.011 (2014).

[b21] Li W., Nicol F. & Szoka F. C. Jr GALA: A designed synthetic pH-responsive amphipathic peptide with applications in drug and gene delivery. Advanced Drug Delivery Reviews 56, 967–985, doi: 10.1016/j.addr.2003.10.041 (2004). 15066755

[b22] Heitz F., Morris M. C. & Divita G. Twenty years of cell-penetrating peptides: From molecular mechanisms to therapeutics. British Journal of Pharmacology 157, 195–206, doi: 10.1111/j.1476-5381.2009.00057.x (2009). 19309362 PMC2697800

[b23] Grasnick D., Sternberg U., Strandberg E., Wadhwani P. & Ulrich A. S. Irregular structure of the HIV fusion peptide in membranes demonstrated by solid-state NMR and MD simulations. European Biophysics Journal 40, 529–543, doi: 10.1007/s00249-011-0676-5 (2011). 21274707

[b24] Morris M. C., Chaloin L., Méry J., Heitz F. & Divita G. A novel potent strategy for gene delivery using a single peptide vector as a carrier. Nucleic Acids Research 27, 3510–3517, doi: 10.1093/nar/27.17.3510 (1999). 10446241 PMC148595

[b25] Varkouhi A. K., Scholte M., Storm G. & Haisma H. J. Endosomal escape pathways for delivery of biologicals. Journal of Controlled Release 151, 220–228, doi: 10.1016/j.jconrel.2010.11.004 (2011). 21078351

[b26] Kwon E. J., Liong S. & Pun S. H. A truncated HGP peptide sequence that retains endosomolytic activity and improves gene delivery efficiencies. Molecular Pharmaceutics 7, 1260–1265, doi: 10.1021/mp1000668 (2010). 20476763 PMC3018701

[b27] Canine B. F., Wang Y. & Hatefi A. Biosynthesis and characterization of a novel genetically engineered polymer for targeted gene transfer to cancer cells. Journal of Controlled Release 138, 188–196, doi: 10.1016/j.jconrel.2009.04.017 (2009). 19379785 PMC2920211

[b28] Sadeghian F., Hosseinkhani S., Alizadeh A. & Hatefi A. Design, engineering and preparation of a multi-domain fusion vector for gene delivery. Int J Pharm 427, 393–399, doi: 10.1016/j.ijpharm.2012.01.062 (2012). 22342333

[b29] Midoux P., Kichler A., Boutin V., Maurizot J. C. & Monsigny M. Membrane permeabilization and efficient gene transfer by a peptide containing several histidines. Bioconjugate Chemistry 9, 260–267, doi: 10.1021/bc9701611 (1998). 9548543

[b30] Mangipudi S. S., Canine B. F., Wang Y. & Hatefi A. Development of a genetically engineered biomimetic vector for targeted gene transfer to breast cancer cells. Molecular Pharmaceutics 6, 1100–1109, doi: 10.1021/mp800251x (2009). 19419197

[b31] Kuil J., Velders A. H. & van Leeuwen F. W. Multimodal tumor-targeting peptides functionalized with both a radio- and a fluorescent label. Bioconjug Chem 21, 1709–1719, doi: 10.1021/bc100276j (2010). 20812730

[b32] Veldhoen S., Laufer S. D., Trampe A. & Restle T. Cellular delivery of small interfering RNA by a non-covalently attached cell-penetrating peptide: Quantitative analysis of uptake and biological effect. Nucleic Acids Research 34, 6561–6573, doi: 10.1093/nar/gkl941 (2006). 17135188 PMC1747183

[b33] Morris M. C., Vidal P., Chaloin L., Heitz F. & Divita G. A new peptide vector for efficient delivery of oligonucleotides into mammalian cells. Nucleic Acids Research 25, 2730–2736 (1997). 9207018 10.1093/nar/25.14.2730 PMC146800

[b34] Simeoni F., Morris M. C., Heitz F. & Divita G. Insight into the mechanism of the peptide-based gene delivery system MPG: Implications for delivery of siRNA into mammalian cells. Nucleic Acids Research 31, 2717–2724, doi: 10.1093/nar/gkg385 (2003). 12771197 PMC156720

[b35] Saleh T., Bolhassani A., Shojaosadati S. A. & Aghasadeghi M. R. MPG-based nanoparticle: An efficient delivery system for enhancing the potency of DNA vaccine expressing HPV16E7. Vaccine 33, 3164–3170, doi: 10.1016/j.vaccine.2015.05.015 (2015). 26001433

[b36] Van Gaal E. V. B. . How to screen non-viral gene delivery systems *in vitro*? Journal of Controlled Release 154, 218–232, doi: 10.1016/j.jconrel.2011.05.001 (2011). 21600249

[b37] Wang Y., Mangipudi S. S., Canine B. F. & Hatefi A. A designer biomimetic vector with a chimeric architecture for targeted gene transfer. Journal of Controlled Release 137, 46–53, doi: 10.1016/j.jconrel.2009.03.005 (2009). 19303038 PMC2925643

[b38] Canine B. F. & Hatefi A. Development of recombinant cationic polymers for gene therapy research. Advanced Drug Delivery Reviews 62, 1524–1529, doi: 10.1016/j.addr.2010.04.001 (2010). 20399239 PMC2939221

[b39] Govindarajan S. . Targeting human epidermal growth factor receptor 2 by a cell-penetrating peptide-affibody bioconjugate. Biomaterials 33, 2570–2582, doi: 10.1016/j.biomaterials.2011.12.003 (2012). 22192536

[b40] Hosseinkhani S. Molecular enigma of multicolor bioluminescence of firefly luciferase. Cellular and molecular life sciences: CMLS 68, 1167–1182, doi: 10.1007/s00018-010-0607-0 (2011). 21188462 PMC11114832

[b41] Torkzadeh-Mahani M., Ataei F., Nikkhah M. & Hosseinkhani S. Design and development of a whole-cell luminescent biosensor for detection of early-stage of apoptosis. Biosensors and Bioelectronics 38, 362–368, doi: 10.1016/j.bios.2012.06.034 (2012). 22770903

[b42] Slama-Schwok A. . Structural changes induced by binding of the high-mobility group I protein to a mouse satellite DNA sequence. Biophysical Journal 78, 2543–2559 (2000). 10777751 10.1016/S0006-3495(00)76799-3 PMC1300844

[b43] Liu F., Frick A., Yuan X. & Huang L. Dysopsonin activity of serum DNA-binding proteins favorable for gene delivery. Journal of Pharmacology and Experimental Therapeutics 332, 500–504, doi: 10.1124/jpet.109.159541 (2010). 19864618 PMC2812122

[b44] Lechardeur D. . Metabolic instability of plasmid DNA in the cytosol: A potential barrier to gene transfer. Gene Therapy 6, 482–497, doi: 10.1038/sj.gt.3300867 (1999). 10476208

[b45] Xiang S. . Uptake mechanisms of non-viral gene delivery. Journal of Controlled Release 158, 371–378, doi: 10.1016/j.jconrel.2011.09.093 (2012). 21982904

[b46] Khalil I. A., Kogure K., Akita H. & Harashima H. Uptake pathways and subsequent intracellular trafficking in nonviral gene delivery. Pharmacol Rev 58, 32–45, doi: 10.1124/pr.58.1.8 (2006). 16507881

[b47] Lühmann T., Rimann M., Bittermann A. G. & Hall H. Cellular uptake and intracellular pathways of PLL-g-PEG-DNA nanoparticles. Bioconjugate Chemistry 19, 1907–1916, doi: 10.1021/bc800206r (2008). 18717536

[b48] Hong S. . The role of ganglioside GM1 in cellular internalization mechanisms of poly(amidoamine) dendrimers. Bioconjugate Chemistry 20, 1503–1513, doi: 10.1021/bc900029k (2009). 19583240 PMC4641442

[b49] Wattiaux R., Laurent N., Wattiaux-De Coninck S. & Jadot M. Endosomes, lysosomes: Their implication in gene transfer. Advanced Drug Delivery Reviews 41, 201–208, doi: 10.1016/S0169-409X(99)00066-6 (2000). 10699315

[b50] Zhao M. & Weissleder R. Intracellular cargo delivery using tat peptide and derivatives. Medicinal research reviews 24, 1–12, doi: 10.1002/med.10056 (2004). 14595670

[b51] Morris M. C., Deshayes S., Heitz F. & Divita G. Cell-penetrating peptides: From molecular mechanisms to therapeutics. Biology of the Cell 100, 201–217, doi: 10.1042/BC20070116 (2008). 18341479

[b52] Rennert R., Neundorf I. & Beck-Sickinger A. G. Calcitonin-derived peptide carriers: Mechanisms and application. Advanced Drug Delivery Reviews 60, 485–498, doi: 10.1016/j.addr.2007.09.008 (2008). 18160173

[b53] Niikura K., Horisawa K. & Doi N. A fusogenic peptide from a sea urchin fertilization protein promotes intracellular delivery of biomacromolecules by facilitating endosomal escape. Journal of Controlled Release 212, 85–93, doi: 10.1016/j.jconrel.2015.06.020 (2015). 26091921

[b54] He H. . Suppression of Hepatic Inflammation via Systemic siRNA Delivery by Membrane-Disruptive and Endosomolytic Helical Polypeptide Hybrid Nanoparticles. ACS Nano 10, 1859–1870, doi: 10.1021/acsnano.5b05470 (2016). 26811880

[b55] Adam S. A. & Geracet L. Cytosolic proteins that specifically bind nuclear location signals are receptors for nuclear import. Cell 66, 837–847, doi: 10.1016/0092-8674(91)90431-W (1991). 1653647

[b56] Gorlich D. & Mattaj I. W. Nucleocytoplasmic transport. Science 271, 1513–1518 (1996). 8599106 10.1126/science.271.5255.1513

[b57] El-Sayed A. & Harashima H. Endocytosis of gene delivery vectors: From clathrin-dependent to lipid raft-mediated endocytosis. Molecular Therapy 21, 1118–1130, doi: 10.1038/mt.2013.54 (2013). 23587924 PMC3677298

[b58] Lai E. & van Zanten J. H. Monitoring DNA/poly-L-lysine polyplex formation with time-resolved multiangle laser light scattering. Biophysical journal 80, 864–873 (2001). 11159453 10.1016/S0006-3495(01)76065-1 PMC1301284

[b59] Artimo P. . ExPASy: SIB bioinformatics resource portal. Nucleic Acids Research 40, W597–W603, doi: 10.1093/nar/gks400 (2012). 22661580 PMC3394269

[b60] Nazari M. & Hosseinkhani S. Design of disulfide bridge as an alternative mechanism for color shift in firefly luciferase and development of secreted luciferase. Photochemical & photobiological sciences: Official journal of the European Photochemistry Association and the European Society for Photobiology 10, 1203–1215, doi: 10.1039/c1pp05012e (2011). 21494742

