# Supplementary material for: Nano-biomimetic carriers are implicated in mechanistic evaluation of intracellular gene delivery
Source: Sci Rep. 2017 Jan 27;7:41507. doi: 10.1038/srep41507 (PMC5269746; doi:10.1038/srep41507)
Supplement: Supplementary Information [file srep41507-s1.pdf]

Nano-biomimetic carriers are implicated in mechanistic evaluation of intracellular gene delivery

Mohsen Alipour[1], Saman Hosseinkhani [2*], Reza Sheikhnejad[3], Roya cheraghi[1]

1. Department of Nano biotechnology, Faculty of Biological Sciences, Tarbiat Modares University, 14115, *Tehran, Iran*

2. Department of Biochemistry, Faculty of Biological Sciences, Tarbiat Modares University, Tehran, 14115, *Iran*

3. Department of Molecular Biology, 13971, *Tofigh Daru Co, Tehran, Iran*

∗ **Corresponding Author**: Prof. Saman Hosseinkhani, Faculty of Biological Science, Tarbiat Modares University, P. O. Box: 14115-175, Tehran, Iran.

Phone: +98-21-82884407; Fax: +98-21-82884718

E-mail address: saman_h@modares.ac.ir

Materials and methods

Desalting, dialysis and determining the concentration

The purified carriers were desalted prior to cellular and structural assays. Briefly, the carrier solutions were dialyzed against phosphate buffered saline (PBS; pH 7.4) or acetate buffer (pH 5.4) at 4 °C for 48 h using a dialysis tubing (Sigma-Aldrich, MWCO 2kDa). The final concentrations of carriers were determined by measuring the UV absorption at 280 nm using a UV-vis spectrophotometer (Biochrom, Cambridge-UK). Carrier stocks were also prepared by addition of glycerol to 10% final concentrations and stored at -20 °C.

Serum stability

Serum stability analysis was performed to evaluate if nanocarriers are protected against serum nucleases. First, HNH and GNH nanocarriers were complexed with 0.5 µg plasmid at N/P ratio of 8, as described above. Then, fetal bovine serum (Invitrogen, CA, USA) at a final concentration of 10 % (v/v) was added to the two batches of nanoparticles and incubated for 1h at 37 °C. Subsequently, SDS was added to the final concentration of 10% to release DNA from nanocarriers. The nanoparticles were then electrophoresed on a agarose gel (1%) and plasmid mobility was visualized by ethidium bromide staining and UV illumination.

Transmission electron microscopy: The HNH and GNH nanocarriers in complex with pGL3 plasmid at N/P ratio of 10 were drop-cast carefully onto a carbon-supported copper grids. The grids were dried in air for 20 min and imaged using Transmission electron microscope (TEM, Zeiss - EM10C - 80 kV).

Atomic Force Microscopy :For observing topography of nanocarriers, 20 µl of HNH and GNH were deposited on mica and spray-dried. The images were acquired on a scanning probe microscope SPM (Veeco Instruments, Sunnyvale, CA, USA) using a Si cantilever in the tapping mode. Minimum image processing was employed and image analysis was done using Picoview software.

FITC labeling of nanocarriers

The HNH and GNH nanocarriers were labeled with Fluorescein isothiocyanate (FITC) according to manufacturer's protocol (Sigma-Aldrich, Wisconsin, USA). Briefly, FITC was dissolved in dimethyl sulfoxide (DMSO) at a final concentration of 1 mg/ml. The 50 µl of this solution was added into 950 µl of each nanocarrier with concentration of 0.5 mg/ml. The mixtures were then vortexed at 37 °C for 2 h and centrifuged at 12000 rpm for additional 2 min. The unbound FITC was separated by dialysis against PBS overnight at 4 °C. Finally, FITC labeled nanocarriers were store at -20 °C.

Structural analysis

Modelling of nanocarriers structures

The three-dimensional structure of nanocarriers were predicted by I-Tasser server [53]. Briefly, the primary sequence of peptide based nanocarriers were deposited on I-Tasser web page. Using this server, the matched regions of sequences were modeled first, based on its similar PDB structure, and then the unmatched regions were produced with an ab initio modeling calculation. The server produced five models, and the best model was selected based its c-score. Finally, the PyMol

software was used to illustrate the 3D structure of models (The PyMOL Molecular Graphics System, Version 1.2r3pre, Schrodinger, LLC).

Fluorescence characterization

The intrinsic fluorescence of HNH and GNH carriers were measured using a Spectrofluorometer (Perkin-Elmer, U.K). The emission spectra of carriers prepared at different pH in PBS and acetate buffers, were recorded over the wavelength ranges of 300-450 nm upon excitation of the tryptophan residues at 295 nm. Furthermore, hydrophobic patches of carriers were investigated by addition of a fluorescence probe, 8-Anilino-1-naphthalenesulfonic acid (ANS; Sigma–Aldrich). The HNH and GNH nanocarriers at 30 μM concentration was incubated with the ANS at 1:30 molar ratio. After 5 min incubation, the emission spectra were recorded between 400 and 700 nm upon excitation at 350 nm.

Structural analysis

CD measurements

The effect of acidic environment on the secondary structure of carriers was investigated by circular dichroism (CD) analysis. The far-UV CD spectra were recorded on a JASCO J-715 spectropolarimeter (Japan) at 25 °C. The CD spectra were obtained from HNH and GNH carriers at a concentration of 0.2 mg/ml, in PBS buffer (pH 7.4) and in acetate buffer (pH 5.4). Spectra were smoothed by noise reduction in JASCO J-715 software. The molar ellipticity [Θ] (degree cm^2 $dmol^{-1}$), was calculated as; $[\theta]\_\lambda = (\theta * MRW * 100)/(cl)$, Where, c is the carrier concentration (g/ml), l is the light path length, θ is measured ellipticity in degrees at wavelength and MRW is the mean residue weight of each carrier.

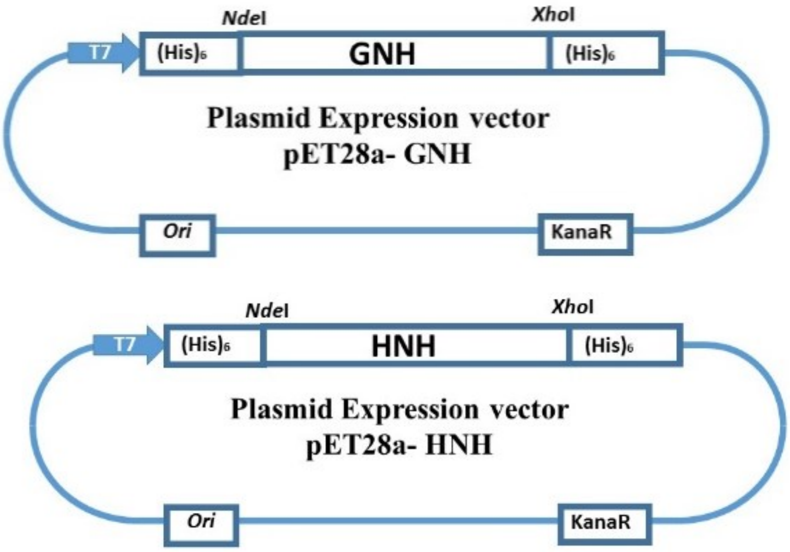

Figures S1.The scheme of HNH and GNH in pET28a expression vector.

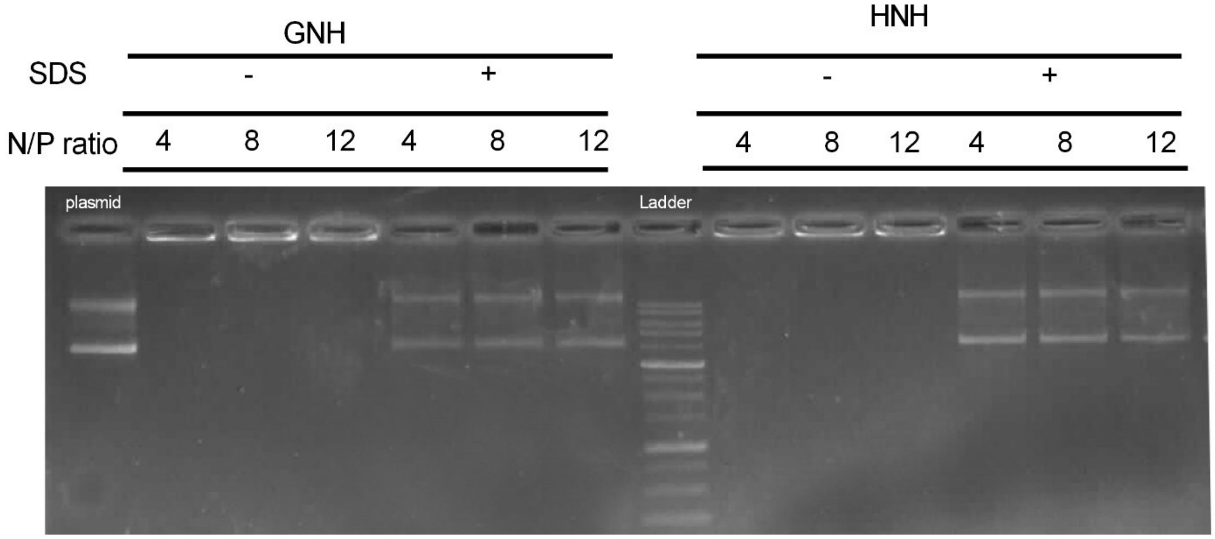

Figures S2. The release of pGL3 plasmid from HNH and GNH nanoparticles.

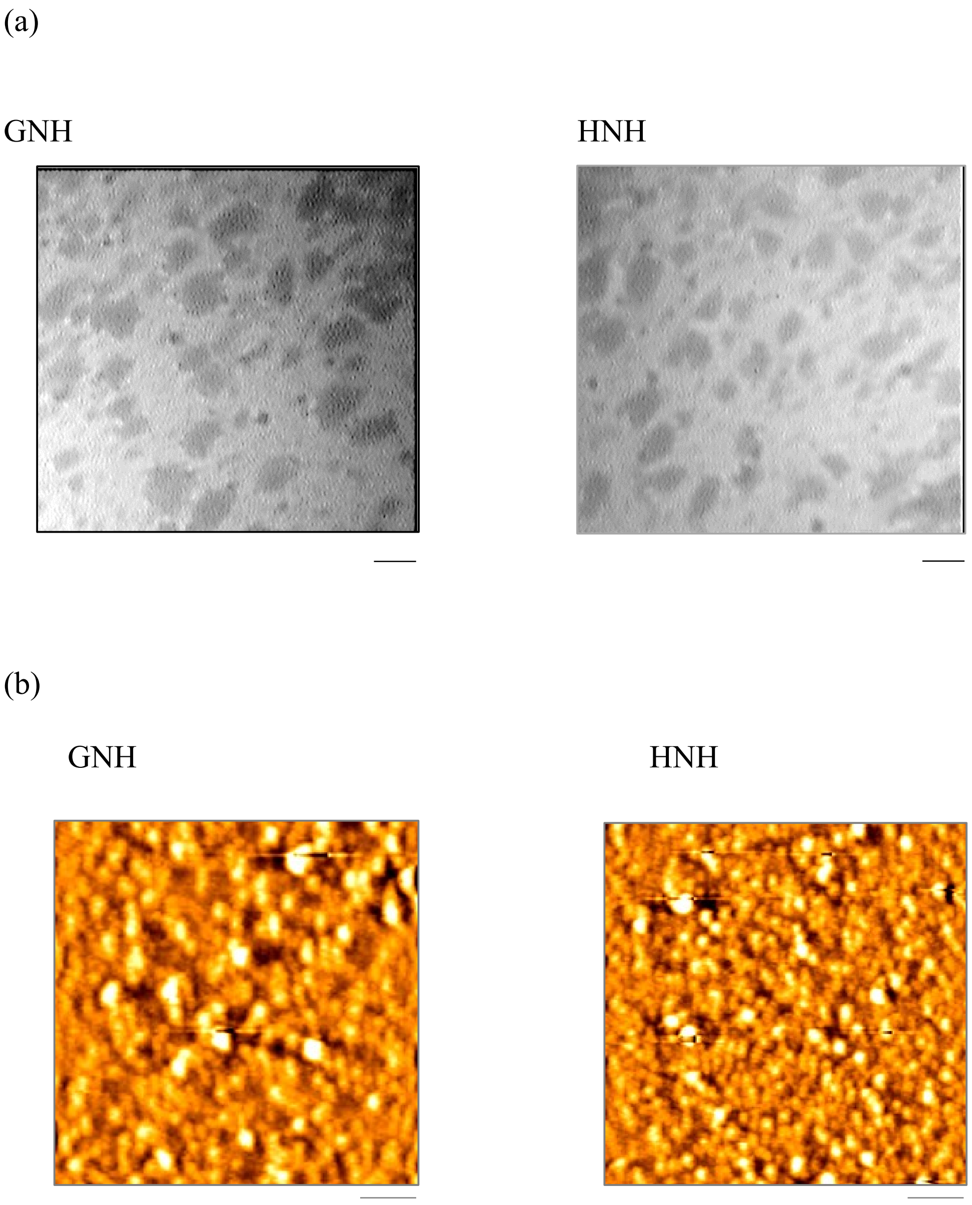

Figures S3. Transmission electron microscopy of nanoparticles and atomic force microscopy of nanocarriers A) TEM of nanoparticles at N/P ratio 10, Scale bar: 100 nm B) Atomic force microscopy image of HNH and GNH nanocarrieres. Scale bar: 20 nm.
